# Efficacy and safety of a balanced salt solution versus a 0.9% saline infusion for the prevention of contrast-induced acute kidney injury (BASIC trial): a study protocol for a randomized controlled trial

**DOI:** 10.1186/s13063-017-2202-2

**Published:** 2017-10-05

**Authors:** Hyung Ah Jo, Sehoon Park, Chan-Duck Kim, Hee-Yeon Jung, Jang-Hee Cho, Ran-hui Cha, Ea Wha Kang, Tae Ik Chang, Sejoong Kim, Hyung-Jong Kim, Byung Ha Chung, Jung Pyo Lee, Jung Tak Park, Seung Hyeok Han, Tae-Hyun Yoo, Dong-Ryeol Ryu, Sung Jin Moon, Jae Hyun Chang, Dong Ki Kim, Kwon Wook Joo

**Affiliations:** 1 Department of Internal Medicine, Seoul National University College of Medicine, Seoul, South Korea; 2 Department of Biomedical Sciences, Seoul National University College of Medicine, Seoul, South Korea; 3 Department of Internal Medicine, Kyungpook National University Hospital, Daegu, South Korea; 4 Department of Internal Medicine, National Medical Center, Seoul, South Korea; 5 Department of Internal Medicine, National Health Insurance Service Ilsan Hospital, Goyang, South Korea; 6 Department of Internal Medicine, Seoul National University Bundang Hospital, Gyeonggi-do, South Korea; 7 Department of Internal Medicine, Bundang CHA Medical Center, CHA University, Gyeonggi-do, South Korea; 8 Department of Internal Medicine, Seoul St. Mary’s Hospital, The Catholic University of Korea, Seoul, South Korea; 9 Department of Internal Medicine, Seoul National University Boramae Medical Center, Seoul, South Korea; 10 Department of Internal Medicine, Yonsei University College of Medicine, Seoul, South Korea; 11 Department of Internal Medicine, Ewha Womans University Mokdong Hospital, Seoul, South Korea; 12 Department of Internal Medicine, Catholic Kwandong University International St. Mary’s Hospital, Incheon, South Korea; 13 Department of Internal Medicine, Gachon University Gil Medical Center, Incheon, South Korea

**Keywords:** Contrast-induced acute kidney injury, Computed tomography, Balanced salt solution

## Abstract

**Background:**

Contrast-induced acute kidney injury (CI-AKI) is one of the most common causes of iatrogenic kidney injury and, therefore, its prevention is an important issue. However, whether the administration of 0.9% saline is the optimal prophylaxis method remains uncertain due to its supra-physiologic chloride component. In particular, recent studies suggest that chloride-restricted solutions showed superiority over 0.9% saline in several clinical settings.

**Methods/design:**

The investigators designed a multicenter randomized controlled trial to compare the efficacy of a balanced salt solution and 0.9% saline in CI-AKI prophylaxis. This study will recruit patients who are scheduled for contrast-enhanced computed tomography (CT) scans with CI-AKI prophylaxis. In this study, participants will be randomized into two study arms; the study group will receive a balanced salt solution, and the control group will receive 0.9% saline. Fluids will be administered as designated in the protocol before and after the CT scan, and an evaluation of baseline clinical status will be performed by obtaining blood and urine samples. During the follow-up visits, the incidence of CI-AKI and long-term outcomes, including the start of renal replacement therapy or all-cause mortality, will be assessed.

**Discussion:**

To our knowledge, this study will be the first study assessing the preventive value of a balanced salt solution over 0.9% saline for CI-AKI. If the trial shows that the balanced salt solution is as effective for CI-AKI prophylaxis as 0.9% saline, the use of the balanced salt solution could be promoted due to the reduced possibility of consequent metabolic acidosis compared to 0.9% saline.

**Trials registration:**

ClinicalTrials.gov, ID: NCT02799368. Registered on 14 June 2016.

**Electronic supplementary material:**

The online version of this article (doi:10.1186/s13063-017-2202-2) contains supplementary material, which is available to authorized users.

## Background

Because iodinated contrast media is widely used in current medicine [1, 2], its well-known side effect, contrast-induced acute kidney injury (CI-AKI), has become one of the most common causes of iatrogenic kidney injury [3, 4]. CI-AKI has been related to increased mortality, longer hospital stays and renal failure in patients with chronic kidney disease (CKD) [3, 5–9]. Hence, preventing CI-AKI has been regarded as an important medical issue [7, 10, 11], and the use of 0.9% saline has been an essential part of CI-AKI prophylaxis [10, 12]. However, 0.9% saline has a pH of approximately 5.5 and contains a supra-physiologic chloride level. Therefore, the use of saline could cause metabolic acidosis, which contributes to renal vasoconstriction [13, 14]. From this clinical point of view, a few trials have tested the efficacy of a sodium bicarbonate fluid solution for CI-AKI prophylaxis, but the results failed to show consistent superiority over 0.9% saline [15]. From another aspect, a recent clinical trial compared the use of 0.9% saline and no prophylaxis for patients with reduced kidney function, and showed no certain benefits with 0.9% saline prophylaxis. However, the effect of CI-AKI prophylaxis may be necessary to be tested in higher-risk patients, such as patients with other risk factors or even lower baseline estimated glomerular filtration rate (eGFR) [16].

Recently, several human studies reported that metabolic acidosis and vasoconstriction are less pronounced when using a balanced salt solution, which has a physiologic level of chloride and a neutral pH, compared to using 0.9% saline [17, 18]. Additionally, there were prospective studies suggesting that using chloride-restrictive solutions, rather than using chloride-rich solutions, for fluid resuscitation can reduce acute kidney injury (AKI) in critically ill patients [19, 20]. In accordance with these findings, a large-scale cohort study was reported, demonstrating the preventive effect of a balanced salt solution for AKI over 0.9% saline [21]. Additionally, one clinical trial is currently recruiting participants to prove the benefit of chloride-restrictive fluids in cardiac surgery [22]. However, to the investigators’ knowledge, there are no ongoing trials regarding the effectiveness of a balanced salt solution for CI-AKI prophylaxis.

This multicenter randomized controlled trial is designed to verify the effectiveness of a balanced salt solution for CI-AKI prophylaxis. After randomization, the study group will receive the balanced salt solution, and the control group will receive 0.9% saline. In this trial, the investigators will address whether the balanced salt solution could be a potential standard solution for CI-AKI prophylaxis.

## Methods/design

**Fig. 1:**
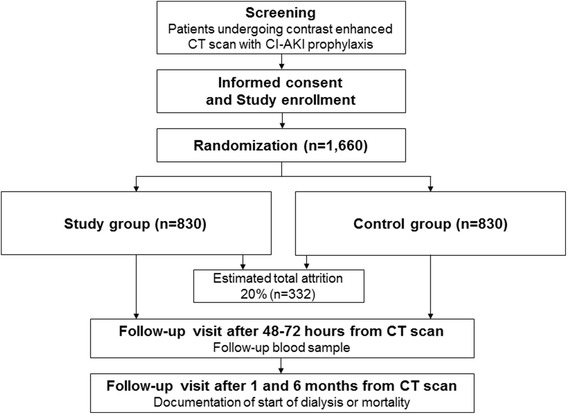
Study flow diagram

**Fig. 2:**
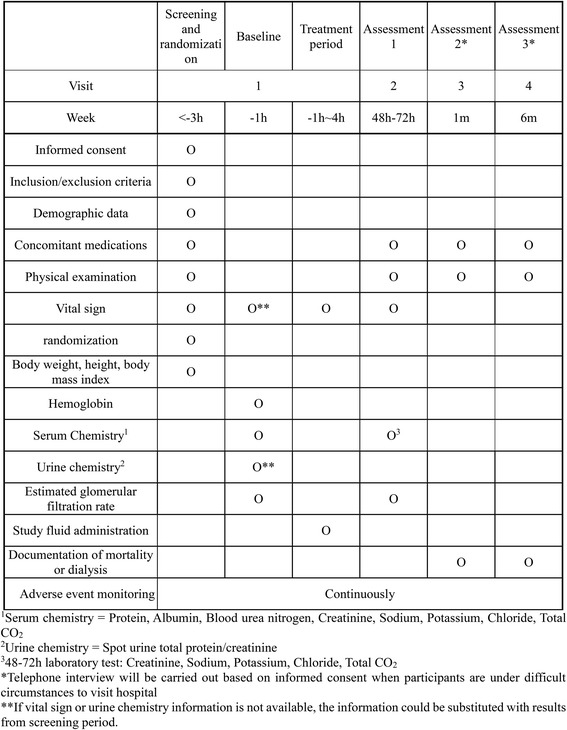
Study timeline and endpoints

The study is a randomized, open-label, active-control, two-parallel-group, multicenter, phase-III study. The study protocol is summarized in Figs. 1 and 2, the latter showing the study timeline which accords with the Standard Protocol Items: Recommendations for Interventional Trials (SPIRIT) Figure. The main goal is to evaluate whether using a balanced salt solution is non-inferior to the use of 0.9% saline for CI-AKI prophylaxis. In current medicine, many clinics provide CI-AKI prophylaxis for patients with decreased kidney function, and this study will recruit these patients who are scheduled for contrast-enhanced computed tomography (CT) scans. After participants are enrolled in the study with informed consent, they will be randomized into the two study arms; the study group will receive a balanced salt solution, and the control group will receive 0.9% saline. The fluids for CI-AKI prophylaxis will be administered as designated in the protocol in the daycare center before and after the CT scan. The baseline clinical status will be evaluated by obtaining blood and urine samples. Next, the participants will be scheduled for a follow-up visit, and their incidence of CI-AKI and further prognosis will be evaluated. The protocol has been designed according to the SPIRIT guidelines (see Additional file 1.)

### Study participants

The study will recruit participants from the following 12 tertiary hospitals in Korea: Seoul National University Hospital, Kyungpook National University Hospital, National Medical Center, National Health Insurance Service Ilsan Hospital, Seoul National University Bundang Hospital, Bundang CHA Medical Center, Seoul St. Mary’s Hospital, Seoul National University Boramae Medical Center, Severance Hospital, Ewha Womans University Mokdong Hospital, Catholic Kwandong University International St. Mary’s Hospital, and Gachon University Gil Medical Center. The planned duration for the study enrollment completion is 2 years. Below, we describe the inclusion and exclusion criteria for the study participants, and the age or eGFR cut-off values are based on a current clinical guideline [11].

Participants’ inclusion criteria are as follows:

1. Adult patients (age 18 years or older) who undergo iodinated contrast-enhanced CT scans
2. Patients with a baseline eGFR, which is measured within 3 months, of less than 45 mL/min/1.73 m^2^ or both having a baseline eGFR less than 60 mL/min/1.73 m^2^ and either one of the following risk factors:
  - diabetes mellitus, or
  - age 60 years or older
3. Patients who are able to provide informed consent and give adequate information for the endpoint assessment

Participants’ exclusion criteria are as follow:

1. Patients with a baseline eGFR of less than 15 mL/min/1.73 m^2^ or who are on dialysis
2. Heart failure with a left ventricular ejection fraction < 45% or severe symptoms (New York Heart Association functional classification III or IV)
3. Co-existing acute pulmonary edema or decompensated heart failure requiring the following medications: dobutamine, dopamine, milrinone, amrinone, or nesiritide
4. Patients with last measured serum potassium level > 5.5 mEq/L or serum sodium level > 145 mEq/L at the screening period or within 3 months before the CT scan
5. History of intravenous or intra-arterial administration of contrast agent within 1 week
6. Previous history of hypersensitivity reaction to the iodinated contrast agent
7. History of multiple myeloma
8. Women who are currently pregnant/breastfeeding or planning pregnancy
9. Patients with an expected survival duration of less than 6 months
10. Patients who are already enrolled in another clinical trial

### Intervention protocols and fluids

The main intervention is the intravenous administration of fluids for CI-AKI prophylaxis. The two types of fluid used in our study are plasma solution A and 0.9% saline. The study fluid, plasma solution A, will be manufactured by CJ HealthCare Corporation, Seoul, South Korea and in facilities following standards of Good Manufacturing Practice. The provided product volume will be 1000 mL, and plasma solution A will contain 5.26 g of sodium chloride, 5.02 g of sodium gluconate, 3.68 g of sodium acetate hydrate, 0.37 g of potassium chloride and 0.3 g of magnesium chloride. In contrast, 0.9% saline will have 9.0 g of sodium chloride with a volume of 1000 mL. The concentrations of each component of the two fluids are summarized in Table 1. Plasma solution A, with 98 mEq/L of chloride, is the balanced salt solution used in this study and will be administered to the study group. Meanwhile, 0.9% saline, which is the standard fluid for CI-AKI prophylaxis and contains 154 mEq/L of chloride, will be used in the control group for CI-AKI prophylaxis. Both fluids will be administered by the designated rate, i.e., 3 mL/kg/h for 1 h before and 1.5 mL/kg/h for 4 h after the CT scans. Participants will remain fasting during the fluid administration. All CT scans will use iso- or low-osmolar contrast agents. *N*-acetylcysteine will not be administered to the participants in this study as this medication lacks a definite benefit for CI-AKI prophylaxis [23]. Other medication use will not be restricted in the study.

**Table 1:** Components of the two fluids used in the study

	0.9% saline	Plasma solution A
Na^+^ (mEq/L)	154	140
K^+^ (mEq/L)		5
Ca^2+^ (mEq/L)		
Mg^2+^ (mEq/L)		1.5
Cl^−^ (mEq/L)	154	98
Acetate (mEq/L)		27
Gluconate (mEq/L)		23
Osmolarity (mOsm/L)	308	295
pH	6.0	7.4

### Study endpoint

The primary endpoint of the study is the incidence of CI-AKI. The event of CI-AKI will be defined as an increase in the serum creatinine level ≥ 0.5 mg/dl or ≥ 25% from baseline at 48–72 h after the CT scan [24]. For the assessment of the primary endpoint, the patients will visit the study hospital and a follow-up blood sample will be drawn; along with other tests, serum creatinine will be measured by the method that has been standardized to isotope dilution mass spectrometry. Next, two secondary endpoints will be evaluated in the study; one is the eGFR decrement at 48–72 h after the CT scan, and the other is the start of dialysis, or mortality, which will be assessed at 1 and 6 months, respectively. The eGFR decrement will be assessed by the baseline and follow-up eGFR values calculated by the serum creatinine as measured in the primary endpoint assessment. The eGFR will be calculated using the Modification of Diet in Renal Disease (MDRD) equation [25]. For documentation of mortality and the beginning of dialysis, the study participants will be questioned via a direct visit or phone poll.

### Sample size

The incidence of CI-AKI when using 0.9% saline for prophylaxis was predicted according to the previous prospective study (11.5%) [26]. In contrast, there was no study regarding the incidence of CI-AKI after the use of a balanced salt solution; therefore, the expected incidence of CI-AKI in the study group was derived from the prospective study, which compared the incidence of AKI in an intensive care unit population according to the resuscitation fluid use (8.4%) [20]. With a non-inferiority limit of 1.5%, a total of 1660 study participants (830 in each group) would result in a power of at least 80% with a one-sided type-1 error rate (*α*) of 2.5%, allowing a 20% withdrawal rate in each group.

### Blinding and randomization

The study is an open-label study; therefore, the type of administered fluid will be disclosed both to the investigators and the participants. The participants and their information will be assigned a unique identifier number at the time of initial enrollment and stored in a web-based data collection system. The group allocation will be performed after randomization in a 1:1 manner, at least 3 h before contrast material administration. The randomization scheme will be generated by using the on-line randomization service developed by Sealed Envelope Ltd. (www.sealedenvelope.com).

### Statistical analysis

All primary and secondary endpoints and serious adverse effects will be analyzed by the investigators at the participating hospitals. The final analysis will be completed at 6 months after the last participant’s trial. The primary endpoint, i.e., the incidence of CI-AKI in the study and control groups will be compared by a non-inferiority test with 1.5% as the non-inferiority margin. The eGFR decrement, one of the secondary endpoints in our study, will be evaluated by chi-squared test. End-stage renal disease (ESRD) progression and mortality will be assessed by Kaplan-Meier survival curve with a log-rank test.

### Data management

All participants’ information related to the study will be recorded in the Case Reporting Format (CRF) and recorded in an electronic, password-protected database. Study participants will only be recognized by their study ID, and their personal identifier will not be recorded and stored. All records will be accessed by the investigators and authorized personnel only to secure confidentiality. The investigators at each participating hospital will monitor the completeness of the CRF. The database will be locked and maintained for 10 years only for the purpose of a secondary analysis or investigations by the attending Institutional Review Boards (IRBs) and the Korean Ministry of Food and Drug Safety (MFDS).

### Adverse events

During the entire study period and after 30 days from the end of the trial, adverse events (AE) will be reported and recorded in the participants’ CRF. The severity of AEs will be graded as mild, moderate and severe, and their relationship between the study groups will also be assessed by clinical judgement. If an AE requiring hospitalization or causing a medically critical situation occurs, the event will be recorded as a severe adverse event (SAE). All SAEs will be reported to the investigator of the attending hospital and the IRB of reference within 24 h after the information has been collected. AEs that cannot deny a relationship to the study must be followed until the AEs have been resolved. Whenever AEs progress to the level of SAEs, the events must also be reported according to the above protocol. At the time of the study result submission, AEs and their relationship to the study will be documented in a table and submitted.

## Discussion

Prophylaxis for CI-AKI is an important clinical issue because CI-AKI is common and worsens patient prognosis. Although the use of 0.9% saline has been the standard method for CI-AKI prophylaxis, its potentially harmful effect was addressed in previous studies. There were several efforts to find a more appropriate infusion fluid for CI-AKI prophylaxis, but trials with sodium bicarbonate fluid failed to demonstrate prophylactic efficacy. The investigators considered the balanced salt solution to be a potential fluid, which can replace 0.9% saline, due to the balanced salt solution’s physiologic components and evidence from recent study results. To our knowledge, the study will be the first study assessing the preventive value of a balanced salt solution over 0.9% saline for CI-AKI. If the proposed trial demonstrates that the balanced salt solution is effective for CI-AKI prophylaxis, the use of the fluid could be promoted due to its reduced likelihood of inducing metabolic acidosis. Therefore, the results of this trial could be useful to improve the prophylaxis method and, consequently, decrease the incidence of CI-AKI and its adverse outcomes.

### Trial status

The BASIC randomized controlled clinical study has received governance approval and is registered at ClinicalTrials.gov (NCT02799368). The trial started recruitment in November, 2016.

## Additional file

Additional file 1: SPIRIT Checklist for the present study protocol. (DOC 113 kb)

## Abbreviations

AE: Adverse event
AKI: Acute kidney injury
CI-AKI: Contrast-induced acute kidney injury
CKD: Chronic kidney disease
CRF: Case Reporting Format
CT: Computed tomography
eGFR: Estimated glomerular filtration rate
IRB: Institutional Review Board
MFDS: Ministry of Food and Drug Safety
SAE: Severe adverse event
